# Reprogramming adipose mesenchymal stem cells into islet β-cells for the treatment of canine diabetes mellitus

**DOI:** 10.1186/s13287-022-03020-w

**Published:** 2022-07-28

**Authors:** Pengxiu Dai, Guixiang Qi, Haojie Xu, Mingde Zhu, Jiakai Li, Yijing Chen, Luwen Zhang, Xinke Zhang, Yihua Zhang

**Affiliations:** 1 Shaanxi Branch of National Stem Cell Engineering and Technology Centre, College of Veterinary Medicine, Northwest A&F University, Yangling, 712100 Shaanxi China; 2 Baiopai (Tianjin) Biotechnology Co., LTD, Jinnan District, Tianjin, 300350 China

**Keywords:** aMSCs, Ra-βCs, Immunogenicity, Cell transplantation therapy, Canine diabetes mellitus

## Abstract

**Background:**

Islet transplantation is an excellent method for the treatment of type I diabetes mellitus. However, due to the limited number of donors, cumbersome isolation and purification procedures, and immune rejection, the clinical application is greatly limited. The development of a simple and efficient new method to obtain islet β-cells is a key problem that urgently requires a solution for the treatment of type I diabetes mellitus.

**Methods:**

In this study, *Pbx1*, *Rfx3*, *Pdx1*, *Ngn3*, *Pax4* and *MafA* were used to form a six-gene combination to efficiently reprogram aMSCs (adipose mesenchymal stem cells) into ra-βCs (reprogrammed aMSCs-derived islet β-cells), and the characteristics and immunogenicity of ra-βCs were detected. Feasibility of ra-βCs transplantation for the treatment of diabetes mellitus in model dogs and clinical dogs was detected.

**Results:**

In this study, aMSCs were efficiently reprogrammed into ra-βCs using a six-gene combination. The ra-βCs showed islet β-cell characteristics. The immunogenicity of ra-βCs was detected and remained low in vitro and increased after transplantation. The cotransplantation of ra-βCs and aMSCs in the treatment of a model and clinical cases of canine diabetes mellitus achieved ideal therapeutic effects.

**Conclusions:**

The aMSCs were efficiently reprogrammed into ra-βCs using a six-gene combination. The cotransplantation of ra-βCs and aMSCs as a treatment for canine diabetes is feasible, which provides a theoretical basis and therapeutic method for the treatment of canine diabetes.

**Supplementary Information:**

The online version contains supplementary material available at 10.1186/s13287-022-03020-w.

## Introduction

A gradual decrease in the number and function of islet β-cells is the main factor involved in the pathogenesis of type I diabetes mellitus. Therefore, replenishing new islet β-cells and rebuilding the insulin secretion system are the key approaches for the treatment of type I diabetes mellitus. Islet transplantation is an excellent method for the treatment of type I diabetes mellitus, but its clinical application is greatly limited due to the limited number of donors, the cumbersome isolation and purification of islets, and immunological rejection [1, 2]. Therefore, the search for cell transplantation methods to replace islets in the treatment of diabetes mellitus has become a hot spot. Adipose mesenchymal stem cells (aMSCs) do not have ethical issues and are easy to separate and culture, have strong proliferation, differentiation, immunoregulatory and immunosuppression abilities after transplantation, promote tissue repair, and are one of the ideal cell resources for the treatment of type I diabetes mellitus [3, 4]. aMSCs can be induced to differentiate into insulin-producing cells (IPCs) in vitro by simulating the developmental environment of islet β-cells and changing the composition of the medium. However, complicated induction procedures and harsh conditions easily cause cell damage, induction costs are high and require multiple cytokines, small-molecule compounds and signaling pathway activators/inhibitors in combination, and the induction effect is not ideal. Therefore, the development of a simple and efficient new method to obtain islet β-cells is a key problem that urgently requires a solution for the treatment of type I diabetes mellitus.

In our previous study, we found that *Pbx1* and *Rfx3* are novel functional genes that regulate the development of aMSCs into islet β-cells [5]. At the same time, four cascades regulating key genes (*Pdx1*, *Ngn3*, *Pax4* and *MafA*) that mediate the development of pancreatic progenitor cells into islet β-cells were screened. In the present study, aMSCs were efficiently reprogrammed into islet β-cells (reprogrammed aMSCs-derived islet β-cells, ra-βCs) using a six-gene combination composed of *Pbx1*, *Rfx3*, *Pdx1*, *Ngn3*, *Pax4* and *MafA*. The immunogenicity of ra-βCs was detected, and ra-βCs and aMSCs were cotransplanted to treat a canine type I diabetes mellitus model and in the clinic, which achieved ideal therapeutic effects, providing a theoretical basis and therapeutic method for the treatment of canine type I diabetes mellitus.

## Materials and methods

### Preparation of adenovirus particles coexpressing multiple genes

Canine *Pbx1* (GeneID: 488669), *Rfx3* (GeneID: 476339), *Pdx1* (GeneID: 493994), *Ngn3* (GeneID: 489022), *Pax4* (GeneID: 482268) and *MafA* (GeneID: 100684427) genes were synthesized by Wuhan Jinkarui Co., Ltd. after codon optimization, and different 2A sequences were connected at the end of each gene (see Additional file 1). According to the instructions of the In-Fusion recombinant cloning kit (Takara, Japan), the target genes were connected to the linear *pAdTrack-CMV* adenovirus shuttle vector (restriction enzyme sites were *BglII* and *HindIII*), and the successfully constructed adenovirus shuttle vector was recombined with *pAdEasy-1* in *E. coli BJ5183* to construct the *pAdEasy-Pbx1-Pdx1-Ngn3-Pax4* (GFP probes) adenovirus vector coexpressing multiple genes. Using *RedTrack-CMV* adenovirus shuttle vectors as mediators (restriction enzyme sites were *BglII* and *HindIII*), the same method was used to construct the *pAdEasy-Rfx3-MafA* (RFP probes) adenovirus vector coexpressing multiple genes. The primers used to construct the vector are shown in Additional file 2. Adenovirus packaging, virus titer determination and detection of target gene expression from multigene-coexpressing adenovirus particles were performed in AAV-293 cells [5].

### Reprogramming aMSCs into ra-βCs

Canine aMSCs were isolated, cultured and frozen in our laboratory [5]. Fourth-generation aMSCs were inoculated into 6-well plates at a density of 1 × 10^5^ cells/well. When the cell density was 75%, two adenovirus particles coexpressing multiple genes were added to the petri dish for infection at a multiplicity of infection = 100. The infected cells were screened and subcultured normally, the subculture was stopped when the cells began to round and accumulate into clusters; the culture was continued until 30 days.

After culture, the reprogrammed cells were observed under a microscope, and the time and number of islet-like cell clusters were counted. RT–qPCR was used to detect the expression levels of the *Pdx1*, *MafA*, *Nkx6.1*, *Nkx2.2*, *Pax4*, *Pcsk1*, *Pcsk2* and *Ins* genes in islet-like cells. Unreprogrammed aMSCs were used as a control, and the *Gadph* gene was used as an internal reference gene. Islet-like cell clusters were detected by staining with a DTZ solution (PB9012, Coolaber, China). Insulin and C-peptide levels were qualitatively detected using immunofluorescence assays. A glucose stimulation assay was used to detect insulin and C-peptide secretion by islet-like cells [5].

### Detection of ra-βCs immunogenicity

The expression levels of DLA Class I, DLA Class II, CD4 and CD80 in ra-βCs were detected using flow cytometry. A unidirectional mixed lymphocyte assay [6, 7] was used to observe the proliferation of peripheral blood mononuclear cells (PBMCs) following ra-βCs stimulation. The lymphocytotoxicity test (LCT) [8, 9] was used to detect the toxicity of presensitized canine lymphocytes to ra-βCs. The binding sites of glutamic acid decarboxylase antibody (GADA) and islet cell antibody (ICA) on ra-βCs were detected using immunofluorescence staining. The immune response induced by ra-βCs in vivo was detected by performing a peritoneal inflammatory infiltration test [10, 11].

### Preparation of the canine type I diabetes mellitus model

All the dogs were purchased from Northwest Agriculture and Forestry University Animal Laboratories (Xian, China). All of the dogs were reared, obtained, and housed in accordance with our institute’s laboratory animal requirements, the dogs were kept in cages in a feeding room without purification equipment at a temperature of 18–25 °C, humidity of 40–60%, airflow value of 0.13–0.18 m/s, ventilation rate of 10–20 times per hour, light normal, noise below 60 dB.

In this study, 24 adult dogs with normal glucose tolerance were subjected to an 85% pancreatectomy and 5 mg/kg streptozotocin (STZ) injection [12]. For the first three days after surgery, dogs were deprived of food and water and were administered a nutritional solution intravenously. On the fourth day after surgery, dogs were fed normally and orally with pancreatin enteric-soluble capsules (Vitabol, China). The dogs were administered Vectaxib chewable tablets (Orbiepharm, China, 3 mg/kg/d, for 5 days) and Beylide (Bayer, Germany, 2.5 mg/kg/d, for 5 days) beginning on the first postoperative day. Fasting blood glucose levels were measured, and dogs were weighed every two days. The index of successful modeling was as follows: a blood glucose level greater than 15 mmol/L for 10 consecutive days, and body weight decreased.

### Cell transplantation for the treatment of diabetes mellitus in model dogs

DiI (Biyuntian Organism, China) was used to label ra-βCs and aMSCs, and the labeled cells were transplanted into the residual pancreatic tissue or testis of model dogs. The specific transplantation groups are shown in Table 1. When the abdominal cavity was closed, wound adhesive (Dermafuse, England) was used to promote healing. The wound was covered with a silver ion wound patch (SKIN KANG RUI, Germany). Postoperative care was consistent with the establishment of a canine type I diabetes model.

**Table 1:** Cell transplantation in diabetic mellitus model dogs

Group	Cells	Graft site
aMSCs + ra-βCs (*n* = 4)	5 × 10^6^ ra-βCs/kg + 5 × 10^6^.aMSCs/kg	Remaining pancreatic tissue
aMSCs + ra-βCs (Testicle) (*n* = 4)	5 × 10^6^ ra-βCs/kg + 5 × 10^6^.aMSCs/kg	Testicle
aMSCs (*n* = 4)	5 × 10^6^.aMSCs/kg	Remaining pancreatic tissue
ra-βCs (*n* = 4)	5 × 10^6^ ra-βCs/kg	Remaining pancreatic tissue
PBS (*n* = 3)	PBS	Remaining pancreatic tissue
PBS + Insulin (*n* = 3)	PBS	Remaining pancreatic tissue, and daily subcutaneous injection of norphin N medium-effect insulin

After cell transplantation, fasting blood glucose levels and body weight were measured every three days. After blood glucose levels stabilized, a glucose tolerance test was conducted after a 10–15 h fast; a 50% glucose solution (2 g/kg) was intravenously injected, and blood glucose levels were measured at 0, 20, 40, 60, 80, 100 and 120 min after the infusion. All dogs were tested for GADA and ICA levels using ELISA kits (Enzyme-linked Biotechnology, China).

One dog each was randomly selected from the aMSCs + ra-βCs group, aMSCs + ra-βCs (testicle) group, aMSCs group and ra-βCs group to collect samples from the graft. Tissue sections for HE staining and frozen sections for insulin immunofluorescence staining (Insulin antibodies (1:600; Abcam, UK)) were generated and analyzed by Wuhan Xavier Biotechnology Co., Ltd.

### Cell transplantation for the treatment of clinical canine diabetes mellitus

Cell transplantation was performed on a dog with type I diabetes mellitus (name: Guaiguai) from a Pet Hospital in Xi'an, China and a dog with type I diabetes mellitus (name: Dadan) from a Stray Animal Center in Xi'an, China. The information of the two dogs is provided below.

The first dog, Guaiguai, a female aged 9 years and weighing 7.50 kg, had type I diabetes mellitus. The onset was 5 years, and the fasting blood glucose level was 40.26 mmol/L in the absence of exogenous insulin. Clinical symptoms included excessive drinking and urination, substantial weight fluctuation, ulceration of hind limbs, severe cataracts in both eyes, and skin diseases of the limbs and trunk (depilation and itching). Since the onset of the disease, the patient was treated with insulin (medium-effect insulin from Nopeace ^N^, 6 IU in the morning and 6 IU in the evening). During treatment, fasting blood glucose levels did not remain stable and fluctuated substantially, and the fasting blood glucose recorded on the next day ranged from 15.15 to 25.35 mmol/L.

The second dog was Dadan, a male aged 5 years and weighing 8.20 kg, with type I diabetes mellitus. At 1 year since onset, the fasting blood glucose level was 25.17 mmol/L without exogenous insulin. Clinical symptoms included excessive drinking and urination, a slight weight fluctuation, normal skin on trunk and limbs, and normal eyes. Since the onset of the disease, insulin was used for treatment (medium-effect insulin from Nopeace ^N^, 4 IU in the morning and 3 IU in the evening). During the treatment, the fasting blood glucose level remained stable with little fluctuation, and the fasting blood glucose level recorded on the next day ranged from 9.05 to 13.28 mmol/L.

The transplanted cells were a mixture of aMSCs and ra-βCs. The graft site was groin fat. The number of ra-βCs cells transplanted was 5 × 10^6^ ra-βCs cells/kg. The number of aMSCs was consistent with that of ra-βCs.

After cell transplantation, fasting blood glucose levels and body weight were measured every two days. GADA and ICA levels were measured using an ELISA kit (Enzyme-linked Biotechnology, China).

### Statistical analysis

Assays were repeated three times. One-way analysis of variance (ANOVA) was used for the statistical comparisons among groups. The tests were performed using IBM SPSS Statistics 25 software (SPSS Inc., Chicago, IL, USA).

## Result

### Preparation of adenovirus particles coexpressing multiple genes

The adenovirus vectors coexpressing multiple genes were verified by sequencing. The titers of *pAdEasy-Pbx1-Pdx1-Ngn3-Pax4* and *pAdEasy-Rfx3-MafA* were 6.9 × 10^7^ IFU/mL and 8.6 × 10^5^ IFU/mL, respectively. Two different adenovirus particles were used to infect AAV-293 cells, and the expression of target genes was detected using RT–qPCR and Western blotting. The results showed that the target genes were expressed at the mRNA and protein levels after cells were infected with adenovirus particles (Additional file 3, the full-length gels and blots were seen in Additional file 4).

### aMSCs were reprogrammed into ra-βCs

When aMSCs were infected with the two adenovirus particles, the cells showed green and red fluorescence simultaneously, and the infection efficiency was approximately 100%.

**Fig. 1:**
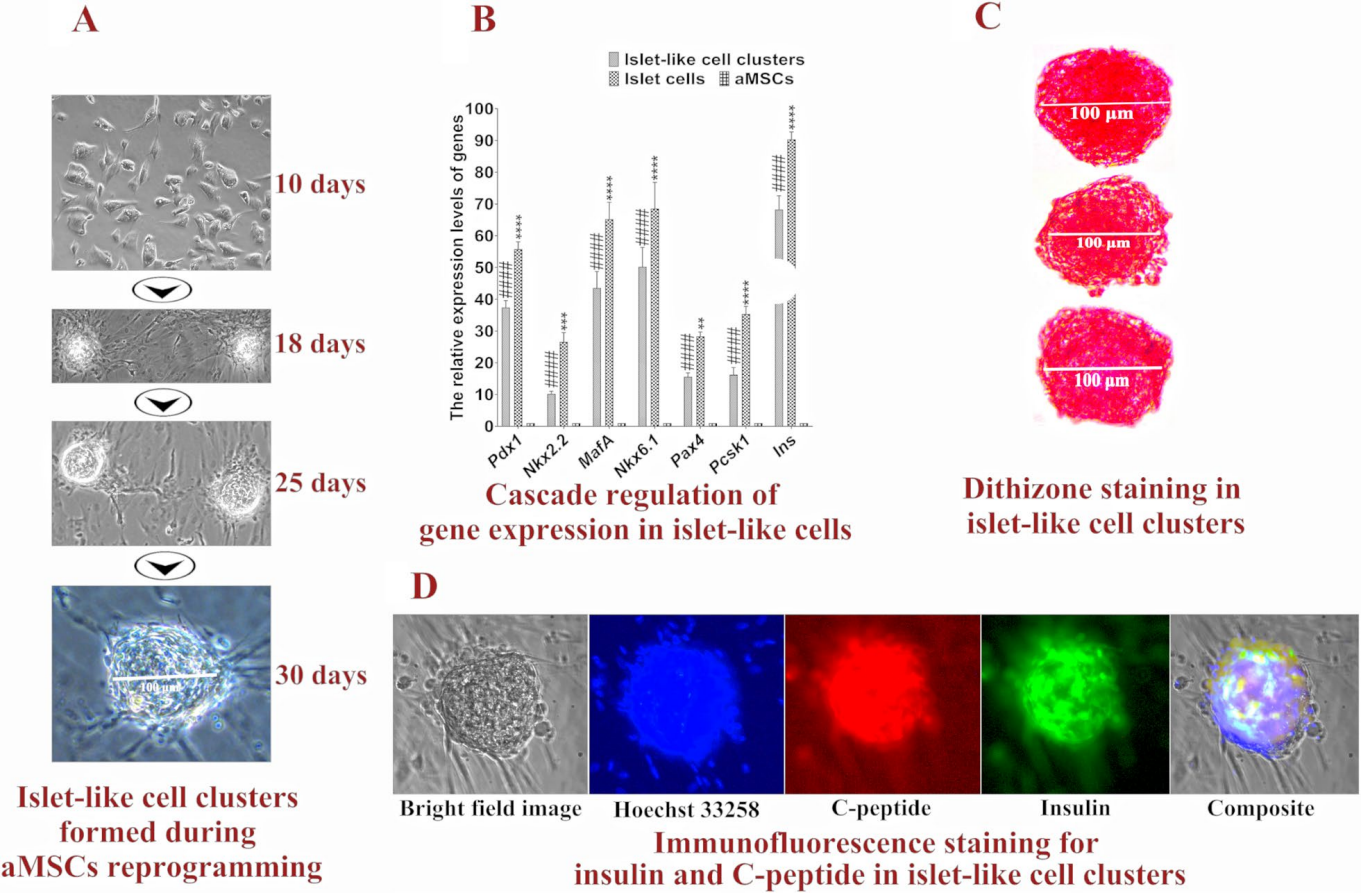
Detection of islet-like cell clusters. **A** Islet-like cell clusters formed during aMSCs reprogramming. **B** Cascade regulation of gene expression in islet-like cells; **P < 0.01 and ****P < 0.0001 represent a very significant difference between islet cells and islet-like cell clusters, ^####^*P* < 0.0001 represents an extremely significant difference between aMSCs and islet-like cell clusters. **C** Dithizone staining in islet-like cell clusters. **D** Immunofluorescence staining for insulin and C-peptide in islet-like cell clusters

**Fig. 2:**
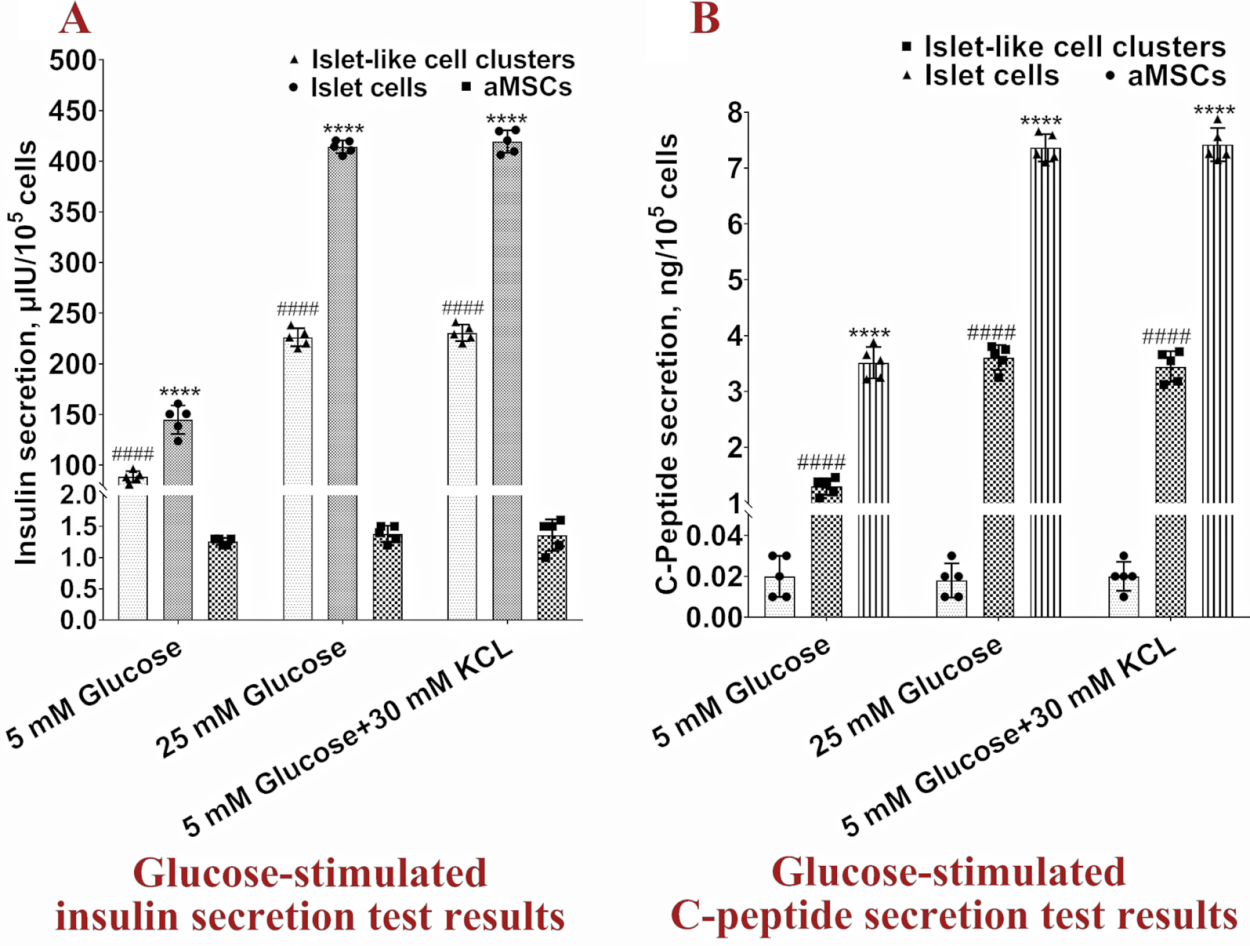
Results of the glucose-stimulated insulin and C-peptide secretion tests. **A** Glucose-stimulated insulin secretion test results. **B** Glucose-stimulated C-peptide secretion test results. ****P < 0.0001 represents a very significant difference between islet cell and islet-like cell clusters, ^####^*P* < 0.0001 represents an extremely significant difference between aMSCs and islet-like cell clusters

After two subcultures for 10 days, the cell morphology changed and began to become round. After culture for 18 days, loose cell clusters formed. After continuous culture for 30 days, the green and red fluorescence disappeared, and the aMSCs formed compact islet-like cell clusters (Fig. 1A). A total of 5 × 10^5^ aMSCs formed 1366 islet-like cell clusters on average (the number of islet-like cell clusters was the total number of cell clusters formed by the initially infected aMSCs after two subcultures), and each cell cluster contained 120 ± 10 cells. The cluster formation rate of aMSCs was 37.57–44.40%. RT–qPCR detection showed significantly higher expression levels of the *Pdx1*, *MafA*, *Nkx6.1*, *Nkx2.2*, *Pax4*, *Pcsk1*, *Pcsk2* and *Ins* genes in islet-like cell clusters than in aMSCs, which reached over 60% of the expression level of mature islet genes in dogs (Fig. 1B). Islet-like cell clusters stained with dithizone appeared scarlet (Fig. 1C). Immunofluorescence assays showed that insulin and C-peptide were expressed in almost all cells in islet-like cell clusters (Fig. 1D). In the glucose-stimulated insulin and C-peptide secretion test, the amount of insulin secreted by islet-like cell clusters under low glucose (5 mM) and high glucose (25 mM) stimulation was 86.75 ± 5.25 μIU/10^5^ cells and 225.80 ± 10.82 μIU/10^5^ cells, respectively, and the amount of C-peptide secreted was 1.30 ± 0.16 ng/10^5^ cells and 3.61 ± 0.19 ng/10^5^ cells, respectively. These values were significantly higher than the levels secreted by aMSCs and reached more than half of the amount secreted by mature canine islet cells (Fig. 2A, B). The islet-like cell clusters obtained in this study were ra-βCs.

### Immunogenicity of ra–βCs

**Fig. 3:**
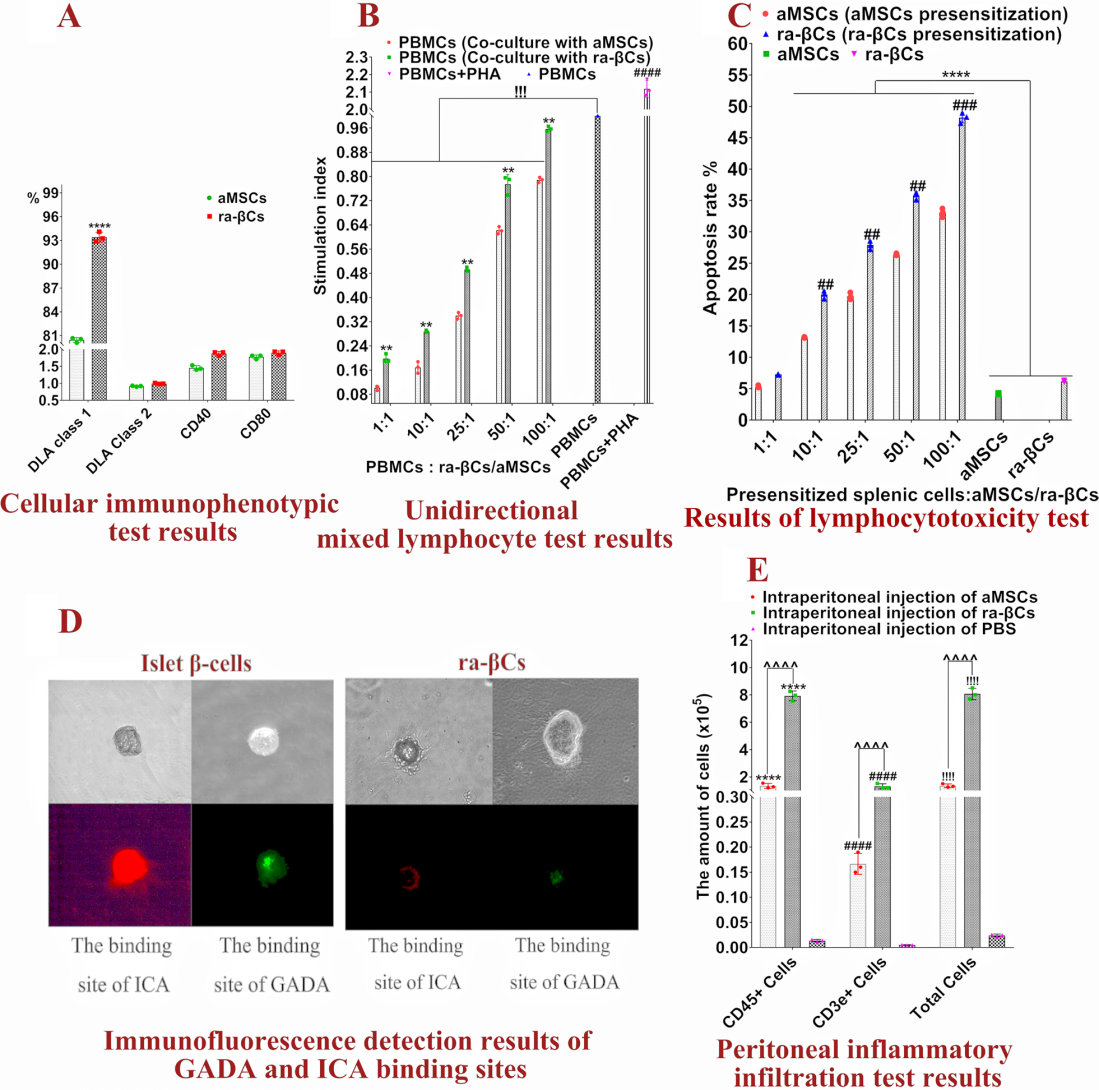
Immunogenicity test results for ra-βCs. **A** Cellular immunophenotypic test results; ****P < 0.0001 represents a very significant difference between aMSCs and ra-βCs. **B** Unidirectional mixed lymphocyte test results; **P < 0.01 indicates an extremely significant difference between PBMSCs (cocultured with aMSCs) and PBMSCs (cocultured with rRa -βCs), ^!!^(*P* < 0.01) and ^!!!!^(*P* < 0.0001) indicated that PBMSCs (co-culture with aMSCs) and PBMSCs (co-culture with ra-βCs) were significantly different from PBMSCs; ^####^(*P* < 0.0001) represents a very significant difference between PBMSCs + PHA and other groups. **C** Results of lymphocytotoxicity test. ^##^(*P* < 0.01) represents the extremely significant difference between aMSCs (co-culture with spleen cells presensitized by aMSCs) and ra-βCs (co-culture with spleen cells presensitized by ra-βCs), ****(*P* < 0.0001) showed extremely significant differences between aMSCs, ra-βCs and other groups. **D** Immunofluorescence detection results of GADA and ICA binding sites. **E** Peritoneal inflammatory infiltration test results, ****(*P* < 0.0001), ^####^(*P* < *0.0001*) and ^!!!!^(*P* < *0.0001*) represented extremely significant difference between intraperitoneal injection of aMSCs, intraperitoneal injection of ra-βCs and intraperitoneal injection of PBS, ^^^^^^(*P* < *0.0001*) represented an extremely significant difference between intraperitoneal injection of aMSCs and intraperitoneal injection of ra-βCs

Regarding the immunophenotype, DLA class I was expressed at significantly high levels in ra-βCs than in aMSCs. Almost no DLA class II, CD40 and CD80 were detected in ra-βCs, and the levels were not significantly different from aMSCs (Fig. 3A), indicating that ra-βCs had low immunogenicity, but higher immunogenicity than aMSCs.

In the unidirectional mixed lymphocyte test shown in Fig. 3B, the stimulation index in the PBMC + PHA group (stimulus index > 2) was significantly higher than that in the other groups, and PBMCs proliferated significantly. When the ratio of PBMCs/ra-βCs was 1:1 to 50:1, ra-βCs significantly inhibited the proliferation of PBMCs. When the ratio of PBMCs to ra-βCs increased to 100:1, no significant difference in the stimulus index was observed compared with the PBMC group (stimulus index = 1). aMSCs significantly inhibited the proliferation of PBMCs compared with ra-βCs. These results indicated that the immunosuppressive function of aMSCs was decreased after reprogramming to ra-βCs, but the proliferation of PBMCs was still inhibited. Adult islet cells did not inhibit the proliferation of PBMCs (Additional file 5).

Presensitized spleen cells were cocultured with aMSCs or ra-βCs in different proportions to observe the rejection of these other cell types by spleen cells. As shown in Fig. 3C, aMSCs and ra-βCs were cultured separately, and cells underwent a low level of self-apoptosis. When aMSCs or ra-βCs were cocultured with presensitized spleen cells at a ratio of 1:1, the apoptosis rate of aMSCs or ra-βCs did not increase significantly, showing that aMSCs or ra-βCs had a certain immune escape ability. However, with the increase in the proportion of presensitized spleen cells, the apoptosis rate of aMSCs or ra-βCs also increased significantly. At the same proportion, the apoptosis rate of ra-βCs was significantly higher than that of aMSCs, suggesting that ra-βCs were more sensitive to presensitized spleen cells than aMSCs.

GADA and ICA levels were detected in 3 dogs diagnosed with type I diabetes mellitus in the Animal Hospital of Northwest Agriculture and Forestry University and Xijing Animal Hospital of Xi'an. Dog No. 1 had the highest GADA level, and dog No. 2 had the highest the ICA level. The corresponding antibodies were purified from the serum of dogs No. 1 and No. 2. The purified antibody was used as the primary antibody for immunofluorescence detection. As shown in Fig. 3D, ICA binding sites were strongly positive and GADA binding sites were weakly positive in canine islet β-cells. In ra-βCs, ICA and GADA binding sites were negative, suggesting that ra-βCs were not affected by GADA and ICA.

After intraperitoneal injection of cells or PBS, the number of CD45^+^ and CD3e^+^ cells was detected. As shown in Fig. 3E, after intraperitoneal injection of PBS, very few CD45^+^ and CD3e^+^ cells were detected. After the injection of aMSCs and ra-βCs, the number of CD45^+^ cells and CD3e^+^ cells was significantly increased, and the number in the ra-βCs group was significantly higher than that in the aMSCs group, suggesting that both aMSCs and ra-βCs induced inflammation and an immune response, but ra-βCs was more likely to induce a response than aMSCs. In addition, after the injection of aMSCs and ra-βCs, the proportions of CD45^+^ cells were 96.24% and 91.44%, respectively, indicating that inflammation was the main reaction induced.

### Preparation of the canine model of type I diabetes mellitus

The pancreatic resection rate of each dog is shown in Additional file 6, and the resection rate was approximately 85%. After resection of some of the pancreas, the STZ solution (5 mg/kg) was injected through the duodenal artery. The two dogs died on the fifth and eighth days after the operation. The 22 dogs had no infection or other postoperative complications after the operation, the stitches were removed on Day 8, and the wound healed. On the eighth day after the operation, some dogs had blood glucose levels greater than 15 mmoL/L, and on the 10th day after the operation, all dogs had blood glucose levels greater than 15 mmoL/L. Over time, the fasting blood glucose level continued to remain > 15 mmoL/L and did not decrease (Additional file 7). With the increase in blood glucose levels, all dogs exhibited polydipsia polyuria symptoms and lost weight (Additional file 7). Since all the dogs were fed pancreatin enteric-coated capsules, all dog feces were normal, and no other abnormalities were observed. The successful establishment of the model in 22 dogs enabled them to be used in the next experiment.

### Cell transplantation for the treatment of diabetes mellitus in model dogs

**Fig. 4:**
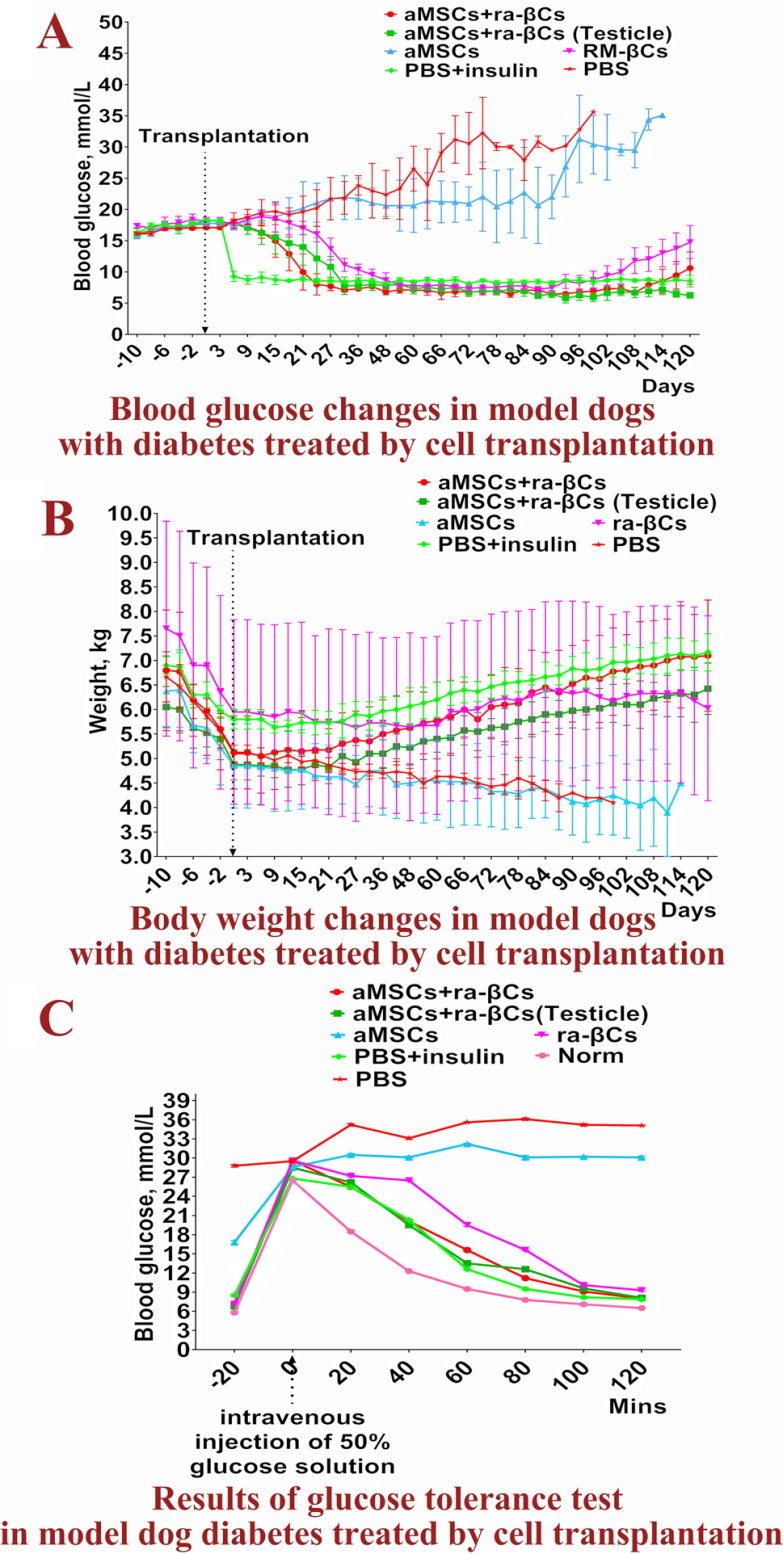
Blood glucose, body weight changes and glucose tolerance test results. **A** and **B** Blood glucose and body weight changes in model dogs with diabetes treated by cell transplantation, -10, -6, -4 and -2 on the horizontal axis indicate the 10, 6, 4 and 2 days before cell transplantation. **C** Results of glucose tolerance test in model dog diabetes treated by cell transplantation, −20 indicates the 20th min before glucose injection

The 22 model dogs with diabetes mellitus were used in the subsequent cell transplantation trial. As shown in Fig. 4A, B, fasting blood glucose levels began to decrease on Day 36 after aMSCs transplantation but did not fall below 15.00 mmoL/L. Fasting blood glucose levels continued to increase to Day 93. The symptoms of polydipsia and polyuria were not alleviated in all dogs, and weight loss continued. Three dogs (one dog was sampled and tested on Day 30) died on Days 103, 112 and 115. In the ra-βCs group, fasting blood glucose levels decreased to less than 8.00 mmol/L on Day 54 after cell transplantation and were maintained for 37 days. With the decrease in fasting blood glucose levels, the symptoms of polydipsia and polyuria gradually decreased until they disappeared, and the body weight increased. On Day 105 after transplantation, blood glucose levels increased to more than 10 mmoL/L and continued to increase over time, with weight loss beginning on Day 117. In the aMSCs + ra-βCs group, fasting blood glucose levels decreased to < 8.00 mmoL/L on Day 24 after cell transplantation and were maintained for 88 days. With the decrease in fasting blood glucose levels, the symptoms of polydipsia and polyuria gradually decreased until they disappeared, and the body weight increased. Blood glucose levels increased after the 111st day after transplantation, and the body weight did not decrease until the 120th day. In the aMSCs + ra-βCs (testicle) group, fasting blood glucose levels decreased to less than 8.00 mmol/L on Day 30 after cell transplantation and were maintained for at least 91 days. With the decrease in fasting blood glucose levels, the symptoms of polydipsia and polyuria were gradually alleviated until they disappeared, and body weight increased. In the PBS group, the fasting blood glucose levels of the three dogs continued to increase, the symptoms of polydipsia and polyuria became increasingly serious, the body weight continued to decrease, the mental state was poor, and they died on the 77th, 88th and 100th days after PBS injection. In the PBS + insulin group, subcutaneous injection of 6 IU (3 IU in the morning and 3 IU in the evening) of insulin Norphin ^N^, a moderately effective insulin treatment, resulted in a stable fasting blood glucose level of 8.00–9.00 mmoL/L the next day and weight gain. On Day 103 after transplantation, the daily insulin dosage was increased to 8 IU (4 IU in the morning and 4 IU in the evening).

After the blood glucose level was reduced and stabilized, the glucose tolerance test was performed on all dogs. In the aMSCs + ra-βCs group, aMSCs + ra-βCs (testicle) group, ra-βCs group, PBS + Insulin group and Norm group, the blood glucose levels decreased to normal or close to normal levels after 120 min; compared with the Norm group, the glucose clearance rate was slower. However, in the aMSCs group and PBS group, the blood glucose level remained high at all-time points and did not decrease (Fig. 4C).

**Fig. 5:**
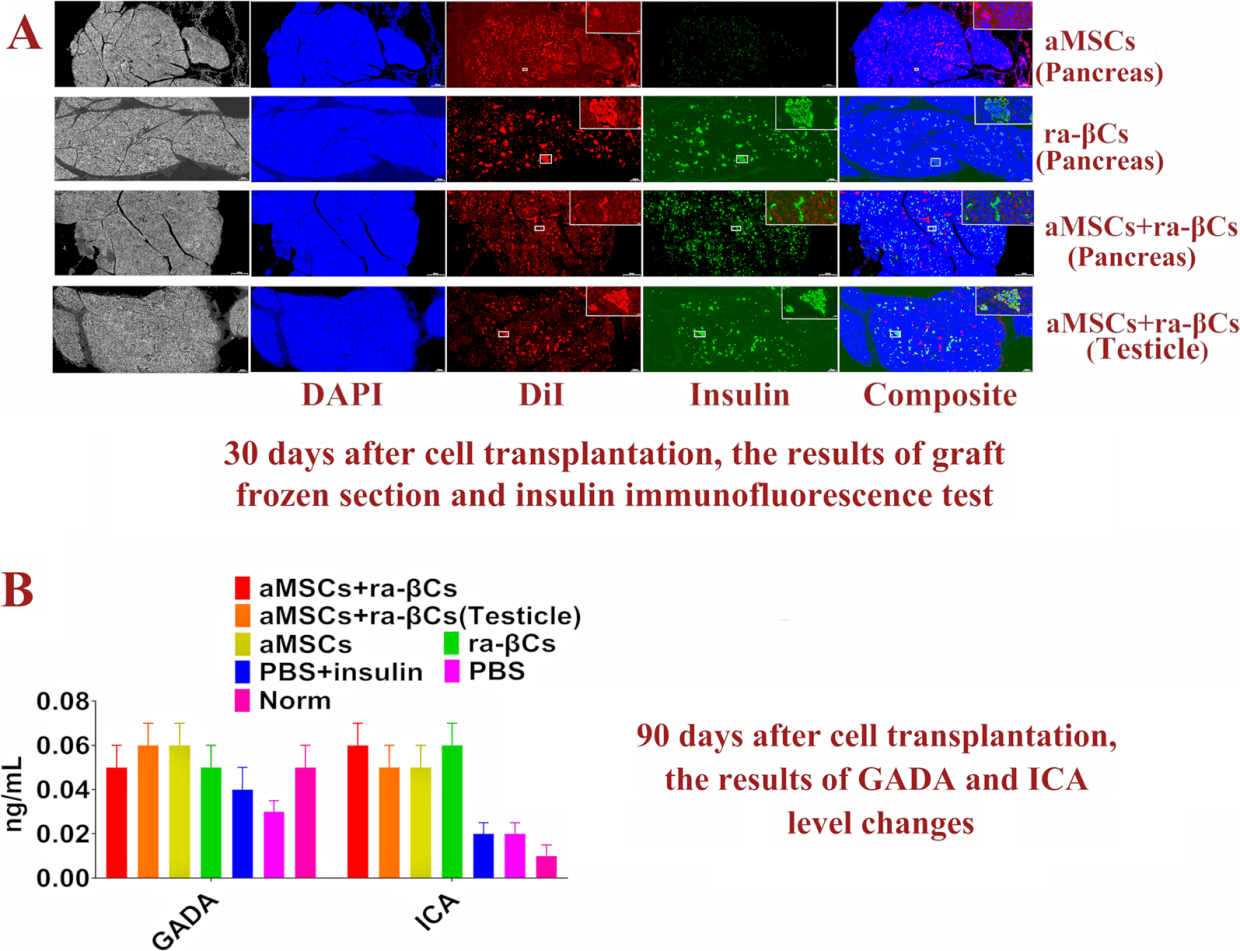
Graft detection and GADA and ICA level test results of the model dog diabetes treated by cell transplantation. **A** 30 days after cell transplantation, the results of graft frozen section and insulin immunofluorescence test, the area in the upper right is a magnified view in the white box. **B** 90 days after cell transplantation, the results of GADA and ICA level changes

One month after cell transplantation, blood glucose levels in the aMSCs + ra-βCs group, aMSCs + ra-βCs (testicle) group and ra-βCs group increased rapidly to > 20.00 mmoL/L after graft removal. The blood glucose level of the aMSCs group remained high after graft removal. Subsequently, four dogs were then euthanized. In the aMSCs group, the transplanted aMSCs (red fluorescence) did not express insulin. In the ra-βCs group, aMSCs + ra-βCs group and aMSCs + ra-βCs (testicle) group, insulin-positive cells were detected, and these cells showed red fluorescence simultaneously, indicating that the insulin-positive cells were transplanted ra-βCs. In the aMSCs + ra-βCs group and aMSCs + ra-βCs (testicle) group, some red fluorescence-positive cells without insulin expression were cotransplanted aMSCs (Fig. 5A).

Ninety days after cell transplantation, serum GADA and ICA levels in all dogs were measured using ELISA kits. The levels of GADA and ICA were very low before and after cell transplantation, and no significant change was observed compared with the normal group, indicating that the transplanted cells did not cause an increase in the levels of these antibodies (Fig. 5B).

**Fig. 6:**
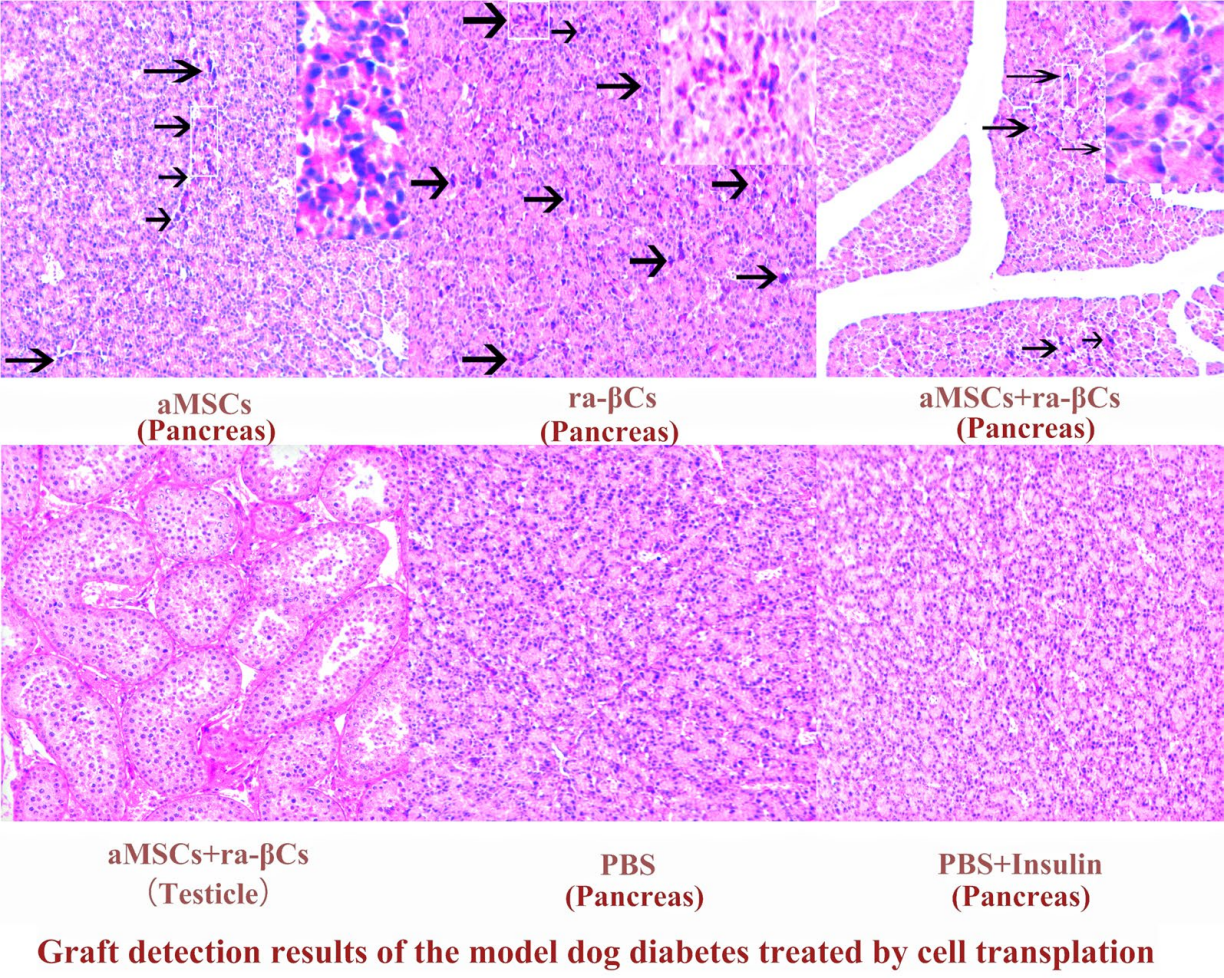
Graft detection results of the model dog diabetes treated by cell transplantation. 120 days after cell transplantation, the graft tissue section and HE staining results. The area in the upper right is a magnified view in the white box, the black arrow points to immune cells

One hundred twenty days after cell transplantation, the dogs were examined for grafting, as shown in Fig. 6. Infiltration of immune cells was observed in the aMSCs + ra-βCs and ra-βCs groups but no immune cells were detected in the aMSCs + ra-βCs (testicle) and PBS + Insulin groups. In the aMSCs group and PBS group, the same test was performed on the grafts after the death of the dogs, and immune cell infiltration was observed in the aMSCs group, while no immune cells were detected in the PBS group. Based on this result, immune cell infiltration occurs at the later stage of cell transplantation, leading to the loss of transplanted cells.

### Cell transplantation for the treatment of clinical canine diabetes mellitus

**Fig. 7:**
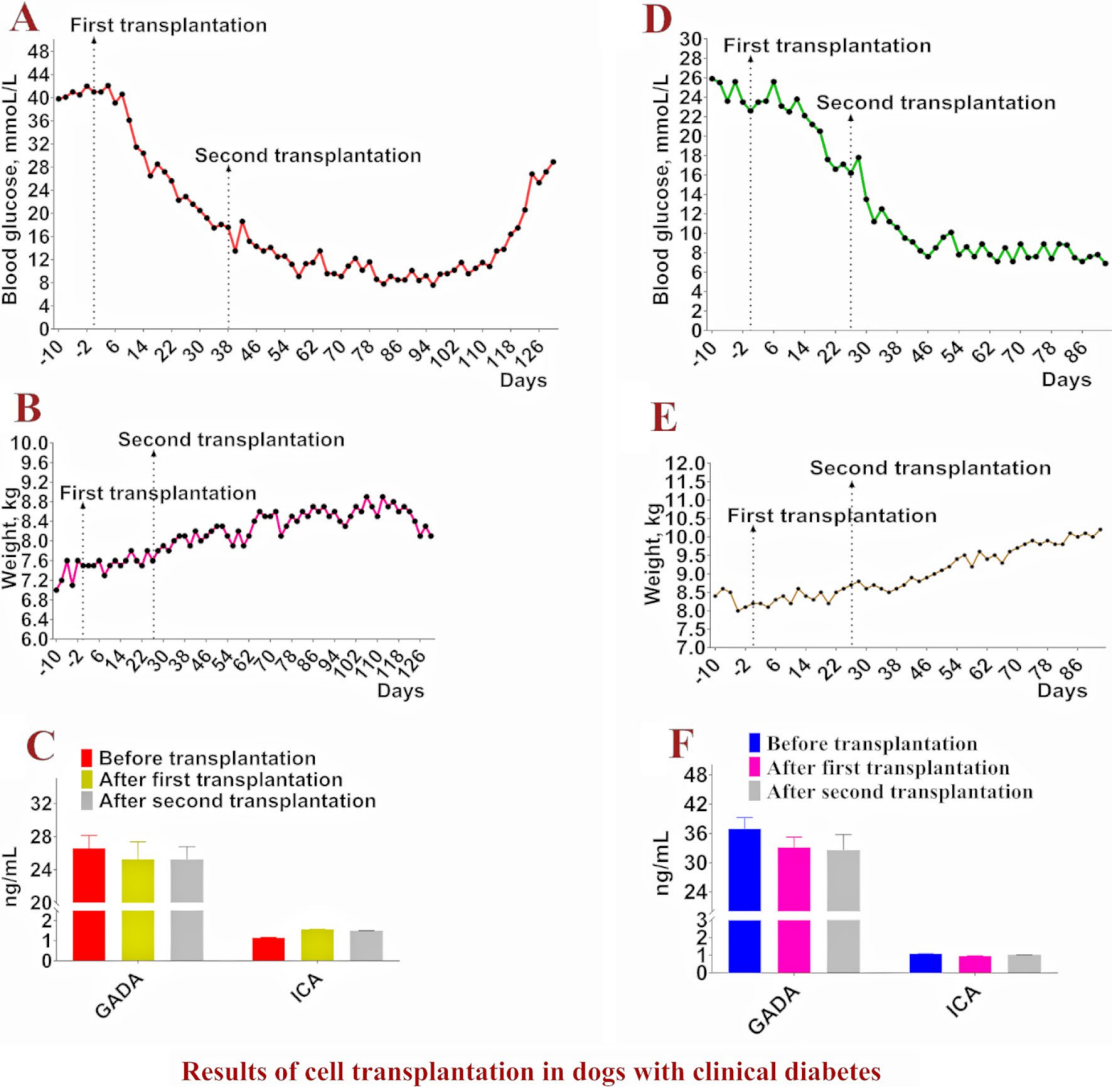
Results of cell transplantation in dogs with clinical diabetes. **A** and **B** Results of blood glucose and body weight changes in the first dog. -10 and -2 on the horizontal axis represent changes in blood glucose and body weight on days 10 and 2 before cell transplantation. **D** and **E** Blood glucose and body weight changes in the second dog. -10 and -2 on the horizontal axis indicate days 10 and 2 before cell transplantation. **C** and **F** Results of changes in GADA ICA levels in first and second dogs at day 20 after the first cells transplant and day 30 after the second cell transplant

The fasting blood glucose level of the first dog showed a decreasing trend after cell transplantation. On Day 34, the blood glucose level decreased to 17.5 mmoL/L and was maintained at this level. On Day 38, a second cell transplantation was performed. Subsequently, the blood glucose level continued to decrease and remained between 7 and 14 mmoL/L, while the exogenous insulin dosage was reduced to 3 IU (1.5 IU in the morning and 1.5 IU in the evening). On the 118th day, the blood glucose level increased to 16.40 mmoL/L and began to increase continuously, and the amount of exogenous insulin required increased (Fig. 7A). During treatment, the body weight increased with the decrease in fasting blood glucose levels, but on the 122nd day, the body weight began to decrease (Fig. 7B). During treatment, the ulceration of the hind limbs healed, and the skin diseases of the limbs and trunk were cured.

The fasting blood glucose level of the second dog showed a decreasing trend after cell transplantation. On Day 20, the blood glucose level decreased to 17.60 mmol/L and remained at this level thereafter. On Day 26, a second cell transplantation was performed. On Day 40, the blood glucose level decreased to less than 10 mmoL/L. In the subsequent period, blood glucose levels fluctuated slightly, basically between 7 and 9 mmoL/L, and the exogenous insulin dosage was reduced to 1 IU (0.5 IU in the morning and 0.5 IU in the evening) (Fig. 7D). Body weight increased as the blood glucose level decreased (Fig. 7E). Unfortunately, the second dog died of an accident on the 93rd day, and no follow-up observation was conducted.

GADA and ICA levels were determined using the same method at 20 days after the first cell transplantation and 30 days after the second cell transplantation. Before and after the first cell transplantation, the contents of GADA and ICA in the serum of the two dogs did not change significantly, especially ICA, which remained at a low level (Fig. 7C, F), indicating that the transplanted cells did not cause an increase in the levels of these two antibodies. After the second cell transplantation, the levels of the two antibodies also showed no significant changes (Fig. 7C, F), indicating that the two antibodies did not bind to the transplanted cells and that the transplanted cells avoided immune clearance mediated by the two antibodies.

## Discussion

In this study, new functional genes, *Rfx3* and *Pbx1,* were introduced to form a six-gene combination based on the cascade of the regulatory genes *Pdx1*, *Ngn3*, *Pax4* and *MafA*. This six-gene combination reprograms aMSCs to ra-βCs, and the secretion of insulin and C-peptide from ra-βCs reached more than half of the levels secreted from β-cells in mature canine islets, which was observed at a high level in similar studies [13–18]. The roles of the new functional genes *Rfx3* and *Pbx1* were highlighted. In addition, aMSCs were subcultured before they differentiated into islet-like cell clusters, which increased the number of ra-βCs, and the cluster rate of aMSCs reached 37.57%–44.40%. The reason why the cluster rate did not reach 100% was that adenovirus particles were diluted during subculture. However, the subculture was positive for the overall acquisition rate of ra-βCs.

In the present study, ra-βCs evaded antigen presentation to a certain extent, had low immunogenicity, and inhibited the proliferation of PBMCs, similar to the reported changes in the immunogenicity of other MSCs after differentiation [19, 20]. Relevant studies have found that islet transplantation mainly activates innate immunity in the early stage, while IPCs transplantation is slow to activate innate immunity, mainly through neutrophil infiltration [21]. In the present study, after the intraperitoneal injection of ra-βCs, the number of CD45^+^ cells was significantly increased, accounting for 91.44% of the total number of ascites cells, indicating that ra-βCs mainly induced neutrophil infiltration in the early stage of the immune response, consistent with the aforementioned studies. Lymphocyte activation and proliferation mediated by inflammatory cytokines and chemokines, as well as the number of CD3e^+^ cells, were also increased very significantly, which may be the main immune response induced after transplantation, but related studies have shown that neutrophil infiltration is attenuated on the third day and the levels of specific inflammatory cytokines and chemokines are either absent or drastically reduced, preventing the migration of immune cells to the transplant site [22]. However, more trials are needed to confirm this finding. ICA and GADA binding sites were not detected on ra-βCs, suggesting that these two antibody-mediated immune responses would not occur after transplantation.

At present, studies on the treatment of diabetes mellitus through the transplantation of IPCs induced by MSCs differentiation are mostly performed in rodent experimental models, and long-term effects are not observed [23–28]. If IPCs treatment for diabetes mellitus is to enter clinical practice, data must be obtained from large experimental animal models of transplant therapy based on the premise of enhancing the maturity of IPCs. Therefore, we used dogs as experimental animals to obtain ra-βCs transplantation data for the treatment of canine diabetes mellitus. In the aMSCs group, higher fasting blood glucose levels were maintained. Under conditions of the pancreatic microenvironment and hyperglycemia, aMSCs differentiate into islet-like cells in vivo, thereby reversing diabetes mellitus [29, 30]. In the present study, this phenomenon did not occur, at least in the early stage of transplantation, according to the blood glucose levels measured in the dogs and the results of graft testing. However, the increasing trend of blood glucose levels in the aMSCs group was slower than that in the PBS group. Differentiation or repair of damaged islet β-cells may occur at a later stage, but the blood glucose level was always high, indicating that even if this situation occurred, it was limited and did not reverse hyperglycemia. In the ra-βCs group, fasting blood glucose levels were maintained at less than 8.00 mmol/L for 37 days, indicating that the transplanted ra-βCs still retained certain immunosuppressive and immunomodulatory functions and did not cause acute rejection. However, as ra-βCs further matured in vivo over time, the immunogenicity was further increased, and immune cells recognized foreign antigens and attacked the transplanted cells. This result was also proven by the presence of immune cell infiltration in the graft on Day 120 after transplantation. In addition, vascular damage, tissue ischemia and macrophage-mediated destruction associated with the immune response may also be important mechanisms underlying ra-βCs disappearance. GADA and ICA levels were not increased in all groups after transplantation, and the loss of transplanted cells was clearly not mediated by these two antibodies. In conclusion, the specific mechanism of ra-βCs disappearance remains to be further elucidated and should be investigated using other techniques. The time at which the blood glucose level decreased in the aMSCs + ra-βCs group was earlier than that in the ra-βCs group, suggesting that aMSCs contributed to the function of ra-βCs. The possible mechanism was that aMSCs promoted ra-βCs colonization and angiogenesis, and the time of ra-βCs functioning was prolonged, indicating that aMSCs possess immunomodulatory, immunosuppressive and nutritional support activities, but aMSCs also induce immune cell infiltration over time. When aMSCs and ra-βCs were cotransplanted into testicles, blood glucose levels were maintained at less than 8.00 mmol/L for at least 91 days, potentially due to the immune privilege of the testicle at the same time that aMSCs performed the functions described above [31]. Immune cells did not infiltrate the testicle in this study. In the glucose tolerance test, the glucose clearance rate of the other groups was slower than that of the normal group, indicating that a certain gap persists between the response of transplanted cells to hyperglycemic stimulation and that of autologous islets, and more research and work are needed to fill this gap.

In the two clinical cases, the treatment effect on the first dog was not as good as that on the model dogs, potentially due to the more complex pathogenesis and internal environment of dogs with clinical diabetes. The fasting blood glucose level of the first dog reached 40.00 mmol/L without the support of exogenous insulin, and thus, this level of hyperglycemia is challenging to reverse through the transplantation of ra-βCs. The second dog responded to treatment better than the first dog, but unfortunately, the second dog died unexpectedly on Day 93 and was unable to be followed further. No significant changes in GADA and ICA levels were detected in these two dogs, indicating that the cotransplantation of ra-βCs and aMSCs did not cause an increase in GADA and ICA levels, and the transplanted cells were not cleared by the immune response mediated by these two antibodies. The clearance mechanism may be similar to or more complex than that of model dogs.

Based on these results, we concluded that ra-βCs obtained in this study reverse hyperglycemia in the body but disappear after a certain period of time, losing the hypoglycemic effect. After cotransplantation with aMSCs, ra-βCs exert a better effect and prolong the time of ra-βCs function. The disappearance of ra-βCs should be mainly mediated by immune clearance or other destructive effects associated with the immune response. Therefore, a better solution to the immune rejection problem will be beneficial for reversing hyperglycemia in the body for a longer time. Immunosuppressive drugs may suppress the immune response of the recipient but have large side effects, such as increased risks of malignant tumors and infection [32]. Recently, many researchers have attempted to reduce the immune response to IPCs derived from MSCs using other techniques, such as completely or selectively knocking out MHC alleles in MSCs or overexpressing PD-L1 [33, 34], protecting the graft from host immune cell infiltration through a physical barrier, such as the TheraCyte encapsulation device system [32, 35, 36] and alginate hydrogel microcapsules [32, 37, 38]; and using 3D bioprinting and nanotechnology to develop encapsulation systems [39, 40]. These physical barriers support islet-like cells to maintain cellular behavior and protect the graft from invasion by innate immune cells. In future studies, these results may be used as a reference to enable transplanted cells to escape immune clearance and increase the time for ra-βCs to function.

## Conclusions

In this study, aMSCs were efficiently reprogrammed into ra-βCs using a six-gene combination (*Pbx1*, *Rfx3*, *Pdx1*, *Ngn3*, *Pax4* and *MafA*). ra-βCs showed islet β-cell characteristics. ra-βCs maintained low immunogenicity in vitro and exhibited increased immunogenicity after transplantation. The cotransplantation of ra-βCs and aMSCs as a treatment for canine diabetes is feasible, which provides a theoretical basis and therapeutic method for the treatment of canine diabetes.

## Supplementary Information

**Additional file 1**. Sequence information of all genes.

**Additional file 2**. PCR primers.

**Additional file 3**. Results of target gene overexpression detection.

**Additional file 4**. Full-length gels and blots.

**Additional file 5**. Adult islet cells did not inhibit the proliferation of PBMCs.

**Additional file 6**. Pancreatic resection rate in dogs.

**Additional file 7**. The changes of blood glucose level and weight.

## Data Availability

Not applicable.

## Abbreviations

aMSCs: Adipose mesenchymal stem cells
ra-βCs: Reprogrammed aMSCs-derived islet β-cells
IPCs: Insulin-producing cells
RT–qPCR: Real-time quantitative polymerasechain reaction
DTZ: Dithizone
PBMCs: Proliferation of peripheral blood mononuclear cells
LCT: Lymphocytotoxicity test
GADA: Glutamic acid decarboxylase antibody
ICA: Islet cell antibody
DLA: Dog leukocyte antigen
STZ: Streptozotocin
