# Supplementary material for: Reprogramming adipose mesenchymal stem cells into islet β-cells for the treatment of canine diabetes mellitus
Source: Stem Cell Res Ther. 2022 Jul 28;13:370. doi: 10.1186/s13287-022-03020-w (PMC9331803; doi:10.1186/s13287-022-03020-w)
Supplement: Supplementary file 1 — Additional file 1. Sequence information of all genes. [file 13287_2022_3020_MOESM1_ESM.docx]

**Sequence information of all genes**

**Name:** Pbx1-F2A**, Length:** 1344 bp
**Base sequence:** ATGGACGAGCAGCCCAGACTGATGCATTCTCATGCCGGCGTCGGAATGGCCGGACATCCTGGACTGTCTCAGCATCTGCAAGATGGCGCTGGCGGAACAGAAGGCGAAGGCGGCAGAAAGCAGGACATCGGAGATATCCTGCAGCAGATCATGACCATCACCGACCAGAGCCTGGATGAGGCCCAGGCCAGAAAACACGCCCTGAACTGCCACAGGATGAAGCCCGCTCTGTTCAACGTGCTGTGCGAGATCAAAGAAAAGACCGTGCTGAGCATCAGAGGCGCCCAAGAGGAAGAACCCACCGATCCTCAGCTGATGAGGCTGGACAATATGCTGCTGGCCGAAGGCGTGGCCGGACCTGAAAAAGGCGGAGGATCTGCAGCCGCTGCTGCTGCCGCTGCAGCTTCTGGCGGAGCTGGCTCTGACAATAGCGTGGAACACAGCGACTACAGGGCCAAGCTGAGCCAGATCAGGCAAATCTACCACACCGAACTCGAGAAGTACGAGCAGGCCTGTAACGAGTTCACCACACACGTGATGAACCTGCTGAGAGAGCAGAGCAGGACCAGGCCTATCAGCCCCAAAGAAATCGAGAGGATGGTGTCCATCATCCACCGGAAGTTCAGCAGCATCCAGATGCAGCTGAAGCAGTCTACCTGCGAGGCCGTGATGATCCTGAGAAGCAGATTCCTGGACGCCAGGCGGAAGAGGCGGAACTTCAACAAACAGGCCACCGAGATCCTGAACGAGTACTTCTACAGCCACCTGAGCAACCCCTATCCTAGCGAGGAAGCCAAAGAGGAACTGGCCAAGAAATGCGGCATCACCGTGTCTCAGGTGTCCAATTGGTTCGGCAACAAGAGGATCCGGTACAAGAAGAACATCGGCAAGTTCCAAGAAGAGGCCAACATCTACGCCGCCAAGACAGCCGTGACAGCCACCAATGTGTCTGCCCACGGCAGCCAGGCCAACTCTCCTAGCACACCTAATAGCGCCGGCAGCTCCAGCAGCTTCAACATGAGCAATAGCGGCGACCTGTTCATGGGCGTGCAGAGCCTGAATGGCGACTCTTACCAAGGCGCTCAAGTGGGCGCCAATGTGCAGAGTCAAGTGGACACCCTGCGGCACGTGATCTCTCAGACCGGCGGATACTCTGATGGACTGGCCGCCTCTCAGATGTACAGCCCTCAGGGCATTTCTGCCAATGGCGGATGGCAGGATGCCACCACACCTAGCTCTGTGACAAGCCCCACTGAAGGCCCTGGCAGCGTGCACTCTGATACCTCTAACAACTTTGACCTGTTAAAGTTGGCGGGAGACGTTGAGTCCAACCCTGGGCCCTGA

**Name:** Rfx3-P2A**, Length:** 2421 bp

**Base sequence:** ATGCAGACCAGCGAAACCGGCTCTGACACCGGCTCTACAGTGACCCTGCAGACAAGTGTGGCCTCTCAGGCTGCTGTGCCTACACAGGTGGTGCAGCAAGTGCCTGTGCAGCAGCAGGTTCAGCAGGTCCAGACAGTGCCTCAGGTGCAGCATGTGTACCCTGCACAGGTGCAGTACGTGGAAGGCAGCGATACCGTGTACACCAACGGCGCCATCAGGACCACCACCTATCCTTACACCGAGACACAGATGTACAGCCAGAACACCGGCGGCAACTACTTCGACACCCAAGGCAGTTCTGCCCAAGTGACCACCGTGGTGTCTAGCCACTCTATGGTCGGAACCGGCGGAATCCAGATGGGAGTGACAGGCGGACAGCTGATCTCTTCTTCTGGCGGCACCTACCTGATCGGCAACAGCATGGAAAACAGCGGCCACAGCGTGACCCACACCACAAGAGCTTCTCCAGCCACCATCGAAATGGCCATCGAGACACTGCAGAAGTCCGACGGCCTGAGCACCCATCGAAGCAGCCTGCTGAATAGCCACCTCCAGTGGCTGCTGGACAACTACGAAACAGCCGAGGGCGTGTCCCTGCCTAGAAGCACCCTGTACAACCACTACCTGCGGCACTGCCAAGAGCACAAGCTGGACCCTGTGAATGCCGCCTCTTTCGGCAAGCTGATCCGGTCCATCTTCATGGGCCTGAGAACCAGAAGGCTGGGCACCAGAGGCAACTCCAAGTACCACTACTACGGCATCAGAGTGAAGCCTGACAGCCCTCTGAACAGGCTGCAAGAGGACATGCAGTATATGGCCATGAGGCAGCAGCCCATGCAGCAGAAGCAGAGGTACAAGCCTATGCAGAAAGTGGACGGCGTGGCCGATGGCTTCACAGGATCTGGACAGCAGACCGGCACCAGCGTGGAACAGACAGTGATTGCCCAGAGCCAGCACCACCAGCAGTTTCTGGATGCCTCTAGAGCCCTGCCTGAGTTCGGCGAGGTGGAAATCAGCTCTCTGCCTGACGGAACCACCTTCGAGGACATCAAGAGCCTGCAGAGCCTGTACAGAGAGCACTGCGAGGCCATCCTGGACGTGGTGGTCAATCTGCAGTTCAGCCTGATCGAGAAGCTGTGGCAGACCTTTTGGAGGTACAGCCCCAGCACACCTACCGACGGAACAACCATCACCGAGAGCAGCAACCTGAGCGAGATCGAGAGCAGACTGCCCAAGGCCAAACTGATCACCCTGTGCAAGCACGAGAGCATCCTGAAGTGGATGTGCAACTGCGACCACGGCATGTATCAGGCCCTGGTGGAAATTCTGATCCCCGACGTGCTGAGGCCCATTCCTTCTGCTCTGACCCAGGCCATCCGGAACTTCGCCAAGTCTCTGGAAGGCTGGCTGAGCAACGCCATGAACAACATCCCTCAGAGGATGATCCAGACCAAGGTGGCCGCCGTGTCTGCCTTTGCTCAGACCCTGAGAAGATACACCAGCCTGAACCATCTGGCCCAGGCCGCTAGAGCTGTGCTGCAGAATACCAGCCAGATCAACCAGATGCTGAACGACCTGAACAGAGTGGATTTCGCCAACGTGCAAGAGCAGGCCTCTTGGGTCTGCCAGTGCGACGACAACATGGTGCAGAGGCTGGAAACCGACTTCAAGATGACCCTCCAGCAGCAGAGCACCCTGGAACAATGGGCTGCCTGGCTGGACAATGTGATGATGCAGGCCCTGAAGCCTTACGAGGGCAGACCTAGCTTTCCCAAGGCCGCCAGACAGTTCCTGCTGAAGTGGTCCTTCTACAGCAGCATGGTCATCAGGGATCTGACCCTCAGAAGCGCCGCCAGCTTCGGATCTTTCCACCTGATCAGACTGCTGTACGACGAGTACATGTTCTACCTGGTCGAGCACAGAGTGGCTCAGGCCACAGGCGAAACACCTATCGCCGTGATGGGCGAAACCTACCTGGTGGACGAGAAGAAAACCCCTATCATGGAACTGTTCATCAGCAAGCTGCTGAACTTCGGCGACCTGAATGCCGTGTCTCCTGGCAACCTGGACAAGGACGAAGGCTCTGAGGTGGAATCTGAGATGGACGAGGAACTGGACGACAGCTCTGAGCCCCAGGCTAAGAGGGAAAAGACCGAGCTGAACCAGGCTTTCCCCGTGGGCTGTATGCAGCCAGTTCTCGAAGGCGGAGTGCAGCCCTCTCTGCTGAATCCTATCCACAGCGAGCACATCGTGACCAGCACACAGACCATCAGGCAGTGTAGCGCCACCGGCAATACCTACACCGCTGTGGTGCATGCCACCTACGCCGAGCTGGATATTCAAGAGACAATCCCCTTCGGCGCCACGAACTTCTCTCTGTTAAAGCAAGCAGGAGATGTTGAAGAAAACCCCGGGCCTTGA

**Name:** MafA-F2A**, Length:** 1092 bp

**Base sequence:** ATGGCTGCCGAACTGGCCATGGGAGCTGAGCTGCCTTCTTCTCCTCTGGCCATCGAATACGTGAACGACTTCGACCTGATGAAGTTCGAAGTGAAGAAAGAGCCTCCTGAGGCCGAGAGGTTCTGCCACAGACTGCCTCCAGGATCTCTGAGCAGCACCCCTCTGAGCACCCCTTGTAGCTCTGTGCCTAGCAGCCCTAGCTTCTGCGCTCCTAGTCCTGGAACAGGCGGAGCTGGTGCTGGTGGTGGCGGCGGAGGCGGAGCACAAGCTGGTGCAGCTGCTGGACCTCCTGGCGGAGGACCTGGTGCTGGCGGAGGTGCTGCTGGAAAACCTGCTCTCGAGGACCTGTACTGGATGAGCGGCTACCCTCAGCATCTGAACCCCGAGGCTCTGCACCTGACACCTGAAGATGCTGTGGATGCCCTGCTCGGCTCTGGACATCATGCTGGTCATCACGGCGCCCATCATCCAGCTGCTGCCGCTGCTTACGAGGCCTTTAGAGGACCAGGATTCGCAGGCGGTGGCGGTGCTGATGAACTCGGAGCTGGACCACCTCATGGCGCTCATCACGCTGCCCACCACCATCATCATGCCGCTCACCATCATCACCACCACCATGCCGCTGCTGCTCATGGCGGAGCAGGACTGCATGTTCGGCTGGAAGAGAGGTTCAGCGACGATCAGCTGGTGTCCATGAGCGTGCGCGAGCTGAACAGACAGCTGAGGGGCTTCAGCAAAGAAGAAGTGATCAGGCTGAAGCAGAAGAGGCGGACCCTGAAGAACAGAGGCTACGCCCAGAGCTGCAGGTTCAAGAGGGTGCAGCAGAGGCACATCCTGGAAAGCGAGAAGTGCCAGCTGCAGAGCCAGGTGGAACAGCTGAAGCTGGAAGTGGGCAGACTGGCCAAAGAGCGCGACCTGTACAAAGAGAAGTACGAGAAGCTGGCCGGCAGAGGCGGTGCCGGATTTCCTAGAGAATCTAGCCCTCCTCAGCCTCAGGCTGGACCTGGCGGTGCAAAAGGCGCCCCTGACTTCTTCCTGAATTTCGACCTGCTCAAACTGGCCGGCGACGTGGAAAGCAACCCTGGACCTTGA

**Name:** Ngn3-P2A**, Length:** 705 bp

**Base sequence:** ATGGCCCCTCATCCTTCTGGTGCTCCTACCGTGCAGGGCACCTACGAAACAGAGAGAAGTTTCCCTGGCGCCTCCGATGATGAAGGCGCCTGTGTTGCTTCTGCCCCTCCATCTCCTGCCAGATCCAGAGGAAATGGCGCCGAGGAAGAAGGCGGAGGTTGTAGGGGAGCCAGCCGGAAACTGAGAACAAGAAGAGGCGGCAGGTCCAGGCCTAAGTCTGAACTGGCCCTGAGCAAGCAGCGGAGAAGCAGAAGAAAGAAGGCCAACGACCGCGAGAGGAACAGGATGCACAACCTGAATAGCGCCCTGGACGCTCTGAGAGGCGTGCTGCCTACCTTTCCTGACGATGCCAAGCTGACCAAGATCGAAACCCTGAGATTCGCCCACAACTACATCTGGGCCCTGACACAGGCCCTGAGGATCGCCGATCACTCTCTGTACGGACTGGAAGCCCCTGCTCTGCCTTGTGGCGAACTGGGATCTCAGGATGGTGGCTCTCCTGGCGATTGGGGCTCTCTGTATAGCCCTGTGTCTCAGGCCGGAAGCCTGTCTCCTGCTGCCAGCCTGGAAGAAAGACCTGGACTGCAGGCTCCAGCCTCTCCTGCATCTCTCAGACCTGGCGCTCTGGCCTTCTCTGATTTCCTGGCCACGAACTTCTCTCTGTTAAAGCAAGCAGGAGATGTTGAAGAAAACCCCGGGCCTTGA

**Name:** Pax4-E2A**,** **Length:** 1197 bp
**Base sequence:** ATGCCTCAAGTCGGATGGGGCGAGGGCAGATGTGTGGACGCCTCTTTTCTGTGCCCTGGCATCAGCAGCGTGAACCAGCTCGGAGGCCTGTTCGTGAATGGCAGACCTCTGCCTCTGGACACCAGGCAGCAGATTGTCAGACTGGCCGTGTCTGGCATGAGGCCCTGCGACATTAGCAGATCCCTGAAGGTGTCCAACGGCTGCGTGTCCAAGATCCTGGCCAGATATTACAGGACCGGCGTGCTGGAACCCAAAGGCATCGGAGGCTCTAAGCCCAGACTGGCTACACCTCCTGTGGTGGCCAGAATCGCTCAGCTGAAAGGCGAGTGCCCTGCTCTGTTTGCCTGGGAGATTCAGAGACAGCTGTGTGCCGAGGGCCTGTGCACCCAGGATAAGACCCCTTCCGTGTCCAGCATCAACAGGGTGCTGAGAGCCCTGCAAGAGGACCAGAGACTGCCTTGGGCTCAGCTCAGATCACCAGCCGTGCTGACCCCTGTGACACACACCCCTCACTCTGGCAGCGAAACCCCTAGAGGACCTCATCCTGGAACCGGCCACCGGAACAGAACCATCTTTAGCCCTGGACAGGCCGAGGCTCTGGAAAAAGAGTTCCAGAGAGGACAGTACCCCGACAGCGTGGCCAGAGGAAAACTGGCCGCTGCTACAAGCCTGCCTGAGGACACAGTCAGAGTGTGGTTCAGCAACAGGCGGGCCAAGTGGCGGAGACAAGAGAAGCTGAAGTGGGAGATGCAGATCAGCGGCGCCTCTCAGGATCTGACCCTGCTGTCTGCTTCTCCCGGAACCACATTCGCCCAGCAGTCTCCAGGATCTGTGCCTACAGCTGTGCCTCCTGCACTGGAATCTCTGGGCCCTAGCTGCTACCAGCTGTACTGGGAGACAAGCCCCGACAGATGCCTGAGGGATACACCTCCACAGGCCAGCCTGAAGCCTTGCTGGGGATATCTGCCTCCTCAACCTAGAAGCCTGGACAGCGTGCTGCTGTGTCACCCCTGTCCTAGCTTCCACTGCCTGCACTACCAGAGCTGGTGCCCTCTGGATCTGGCTCTGGCACAACCTCCTACACCAGCCAAGCCTGGCACCAGAGAAGAAGGCCAGGCCGGCAAAGAAATCGGCCAGTGCACCAATTACGCCCTGCTGAAACTGGCTGGCGACGTGGAAAGCAACCCCGGACCTTGA

**Name:** Pdx1-E2A**, Length:** 909 bp **Base sequence:** ATGAACAGCGAGGAACAGTTCTACGCCGCCACACAGCTGTACAAGGACCCTTGCGCCTTCCAGAGAGGACCCGCTCCTGAGTTTTCTGCCTCTCCTCCTGCCTGTCTGTACATGGGCAGACAGCCTCCACCTCCTCCACCACCTTTTCCAGGCACACTGGGAGCACTGGAACAGGGCTCTCCACCTGACATCAGCCCTTACGAAGTGCCTCCTCTGGCCGACGATTCTGCCGTGGCTCATCTGCACCATCATCTGCCTGCTCAGCTGGCCCTGCCTCATCCACCAGCTGGACCATTTCCAGAAGGCGCCGAACCTGCCGCTCTGGAAGAACCTTCTAGAGTGCAGCTGCCCTTTCCATGGATGAAGTCCACAAAGGCCCACGCCTGGAAAGGACAATGGGCTGGCGGAGCCTATGTGGCCGAGCCTGAGGAAAACAAGAGGACCAGGACCGCCTACACAAGGGCCCAACTGCTGGAACTGGAAAAAGAGTTCCTGTTCAACAAGTACATCAGCAGGCCCCGCAGAGTGGAACTGGCCGTGATGCTGAATCTGACCGAGAGGCACATCAAAATCTGGTTCCAGAACAGGCGCATGAAGTGGAAGAAAGAAGAGGACAAGAAGCGCTCCTGCGGCACAGCTCCTGGCGGAGTTGCTGATGCAGAGCCTGAACAGGATTGCGCCGTGTCTAGCGGAGAGGAACTGCTGGCTCTGCCACCAGCACTGCCTCCAGGCGGAGCTGTTCCTCCTGCTGTGCCTGCTGCTGCTAGAGAAGGCAGACTGCCACCTGGCCTGTCTGCTTCTCCTCAGCCTTCTTCTGTGGCTCCCGTGCGGCCTCAAGAGCCTAGACAGTGTACCAACTACGCCCTGCTGAAACTGGCCGGCGACGTGGAATCTAACCCCGGACCTTGA
