# Supplementary material for: Reprogramming adipose mesenchymal stem cells into islet β-cells for the treatment of canine diabetes mellitus
Source: Stem Cell Res Ther. 2022 Jul 28;13:370. doi: 10.1186/s13287-022-03020-w (PMC9331803; doi:10.1186/s13287-022-03020-w)
Supplement: Supplementary file 2 — Additional file 2. PCR primers. [file 13287_2022_3020_MOESM2_ESM.docx]

**PCR primers for the amplification of Pbx1-Pdx1-Ngn3-Pax4 gene segments**

| Primer name | Primer sequence |
| --- | --- |
| Z-PBX1-F | tgaaccgtcagatccgctagagatctGCCACCATGGACGAGCAGCCCAGACTGA |
| PBX1-PDX1-R | cgtagaactgttcctcgctgttcatGGGCCCAGGGTTGGACTCAACG |
| P1-PDX1-F | agacgttgagtccaaccctgggcccGCCACCATGAACAGCGAGGAACAGTTCT |
| PDX1-NGN3-R | gagcaccagaaggatgaggggccatAGGTCCGGGGTTAGATTCCACG |
| P1-NGN3-F | cgacgtggaatctaaccccggacctGCCACCATGGCCCCTCATCCTTCTGGTG |
| NGN3-PAX4-R | cctcgccccatccgacttgaggcatAGGCCCGGGGTTTTCTTCAACA |
| N3-PAX4-F | agatgttgaagaaaaccccgggcctGCCACCATGCCTCAAGTCGGATGGGGCG |
| PAX4-Z-R | ggatcggatatcttatctagaagcttTCAGCCGATTTCTTTGCCGGCC |

**PCR primers for the amplification of Rfx3-MafA gene segments**

| Primer name | Primer sequence |
| --- | --- |
| Z-RFX3-F | tgaaccgtcagatccgctagagatctGCCACCATGCAGACCAGCGAAACCGGCT |
| RFX3-MAFA-R | cagttcggcagccatAGGCCCGGGGTTTTCTTCAACA |
| R1-MAFA-F | gaaaaccccgggcctGCCACCATGGCTGCCGAACTGGCCATGG |
| MAFA-Z-R | ggatcggatatcttatctagaagcttTCAAGGTCCAGGGTTGCTTTCCACG |
