# Supplementary material for: Reprogramming adipose mesenchymal stem cells into islet β-cells for the treatment of canine diabetes mellitus
Source: Stem Cell Res Ther. 2022 Jul 28;13:370. doi: 10.1186/s13287-022-03020-w (PMC9331803; doi:10.1186/s13287-022-03020-w)
Supplement: Supplementary file 5 — Additional file 5. Adult islet cells did not inhibit the proliferation of PBMCs. [file 13287_2022_3020_MOESM5_ESM.docx]

| PBMCs (Co-culture with adult islet cells ) | | | |
| --- | --- | --- | --- |
| PBMCs : adult islet cells | Stimulation index | | |
| 1:1 | 0.94 | 0.95 | 0.92 |
| 10:1 | 0.97 | 0.95 | 0.92 |
| 25:1 | 0.98 | 0.95 | 0.98 |
| 50:1 | 0.96 | 0.95 | 0.96 |
| 100:1 | 0.98 | 0.98 | 0.96 |
