# Supplementary material for: Reprogramming adipose mesenchymal stem cells into islet β-cells for the treatment of canine diabetes mellitus
Source: Stem Cell Res Ther. 2022 Jul 28;13:370. doi: 10.1186/s13287-022-03020-w (PMC9331803; doi:10.1186/s13287-022-03020-w)
Supplement: Supplementary file 6 — Additional file 6. Pancreatic resection rate in dogs. [file 13287_2022_3020_MOESM6_ESM.docx]

**Pancreatic resection rate in dogs**

| Dog number | Resection ratio (Resection length, Length of right lobe of pancreas) | Resection ratio (Resection length, Length of left lobe of pancreas) |
| --- | --- | --- |
| 1 | 85.71%（9.00 cm，10.50 cm） | 82.35%（7.00 cm，8.50 cm） |
| 2 | 84.61%（11.00 cm，13.00 cm） | 85.00%（8.50 cm，10.00 cm） |
| 3 | 83.33%（10.00 cm，12.00 cm） | 85.00%（8.50 cm，10.00 cm） |
| 4 | 85.71%（9.00 cm，10.50 cm） | 88.88%（8.00 cm，9.00 cm） |
| 5 | 84.21%（8.00 cm，9.50 cm） | 85.71%（9.00 cm，10.50 cm） |
| 6 | 86.36%（9.50 cm，11.00 cm） | 85.71%（9.00 cm，10.50 cm） |
| 7 | 83.33%（10.00 cm，12.00 cm） | 85.00%（8.50 cm，10.00 cm） |
| 8 | 83.87%（13.00 cm，15.50 cm） | 85.00%（8.50 cm，10.00 cm） |
| 9 | 83.33%（10.00 cm，12.00 cm） | 85.71%（9.00 cm，9.50 cm） |
| 10 | 86.36%（9.50 cm，11.00 cm） | 85.71%（9.00 cm，10.50 cm） |
| 11 | 84.61%（11.00 cm，13.00 cm） | 86.36%（9.50 cm，11.00 cm） |
| 12 | 84.21%（8.00 cm，9.50 cm） | 85.00%（8.50 cm，10.00 cm） |
| 13 | 86.95%（10.00 cm，11.50 cm） | 84.00%（10.5 cm，12.50 cm） |
| 14 | 83.33%（10.00 cm，12.00 cm） | 86.95%（10.00 cm，11.50 cm） |
| 15 | 86.95%（10.00 cm，11.50 cm） | 85.00%（8.50 cm，10.00 cm） |
| 16 | 85.71%（12.00 cm，14.00 cm） | 84.61%（11.00 cm，13.00 cm） |
| 17 | 85.18%（11.5 cm，13.50 cm） | 86.36%（9.50 cm，11.00 cm） |
| 18 | 85.71%（12.00 cm，14.00 cm） | 86.36%（9.50 cm，11.00 cm） |
| 19 | 83.33%（10.00 cm，12.00cm） | 85.00%（8.50 cm，10.00 cm） |
| 20 | 83.87%（13.00 cm，15.50 cm） | 84.61%（11.00 cm，13.00 cm） |
| 21 | 86.36%1（9.50 cm，11.00 cm） | 85.00%（8.50 cm，10.00 cm） |
| 22 | 85.00%（8.50 cm，10.00 cm） | 85.71%（9.00 cm，10.50 cm） |
| 23 | 84.21%（8.00 cm，9.50 cm） | 85.00%（8.50 cm，10.00 cm） |
| 24 | 83.33%（10.00 cm，12.00 cm） | 84.21%（8.00 cm，9.50 cm） |
